# Enhancing Safety in Mechanical Ventilation: A Quality Improvement Initiative Targeting Unplanned Extubations in a Tunisian PICU

**DOI:** 10.1097/pq9.0000000000000805

**Published:** 2025-04-02

**Authors:** Farah Thabet, Seyfeddine Zayani, Abir Daya, Chokri Chouchane, Slaheddine Chouchane

**Affiliations:** From the * Pediatric Department, Fattouma Bourguiba University Hospital, Monastir, Tunisia; † School of Medicine, University of Monastir, Monastir, Tunisia.

## Abstract

**Background:**

Unplanned extubation (UE) in pediatric intensive care units (PICUs) is a critical adverse event that can lead to severe complications, including respiratory distress and hypoxia. This study aimed to reduce UE incidence among mechanically ventilated children by implementing targeted quality improvement interventions.

**Methods:**

A quality improvement initiative was conducted in a 7-bed PICU at a university-affiliated hospital in Tunisia from January 2022 to December 2023. The study included three phases: baseline assessment, intervention implementation, and postintervention evaluation. Approaches for improvement included using a key driver diagram and Pareto analysis which led to interventions such as standardized endotracheal tube (ETT) fixation procedures, sedation management, and staff training. The outcome was monitored using statistical process control methods, particularly a U chart to track UE rates.

**Results:**

Following the implementation of the quality improvement interventions, the UE rate decreased from 3.62 to 2.06 per 100 ventilation days, a 42.7% reduction (*P* = 0.015). Statistical process control analysis indicated a statistically significant shift, confirming the effectiveness of the interventions.

**Conclusions:**

Targeted quality improvement interventions, including standardized protocols and staff training, significantly reduced the incidence of UEs in the PICU. These findings underscore the importance of continuous improvement efforts in enhancing patient safety in resource-limited settings.

## INTRODUCTION

The provision of critical care for mechanically ventilated children in a pediatric intensive care unit (PICU) presents complex and important challenges for healthcare professionals. In resource-limited settings like Tunisia, hospitals may face additional challenges such as specialized equipment shortages, staffing variability, frequent reliance on float nurses who are not fully trained PICU nurses, and training resource limitations. These constraints can affect the quality and consistency of care, making quality improvement (QI) initiatives particularly important. Unplanned extubations (UEs), defined as the unintended removal of the endotracheal tube (ETT) before the completion of treatment, represent significant adverse events associated with increased morbidity and mortality. Inadequate management of UEs can result in hypoxia, respiratory failure, prolonged mechanical ventilation, extended ICU stays, glottic and subglottic injury due to mechanical traumas, and increased risk of ventilator-associated pneumonia.^1–4^ Although factors such as patient agitation, nurse-to-patient ratios, and excessive secretions contribute to UEs, implementing targeted QI interventions can mitigate these risks.^5,6^

Our specific, measurable, achievable, relevant, and time-bound (SMART) aim was to decrease the rate of UE by 40% from baseline within 12 months in a Tunisian PICU by implementing a structured QI initiative, thus highlighting unique challenges and successes encountered in a developing healthcare environment.

## METHODS

### Context

We conducted this QI study in a 7-bed PICU at a university-affiliated tertiary care hospital in Monastir, Tunisia. The hospital serves as a regional referral center, providing advanced pediatric care to critically ill children across the region. The PICU is staffed by a multidisciplinary team, including 2 pediatric intensivists, pediatric residents, nurses, 2 respiratory therapists, and support staff, all trained to manage complex medical conditions. However, the unit often relies on floating nurses who may not be permanently assigned to the PICU, leading to potential inconsistencies in care practices and the need for ongoing training. Furthermore, the healthcare environment in Tunisia presents unique challenges, including resource limitations and variability in staffing, which may affect the consistency of care.

### Intervention

**Fig. 1.**
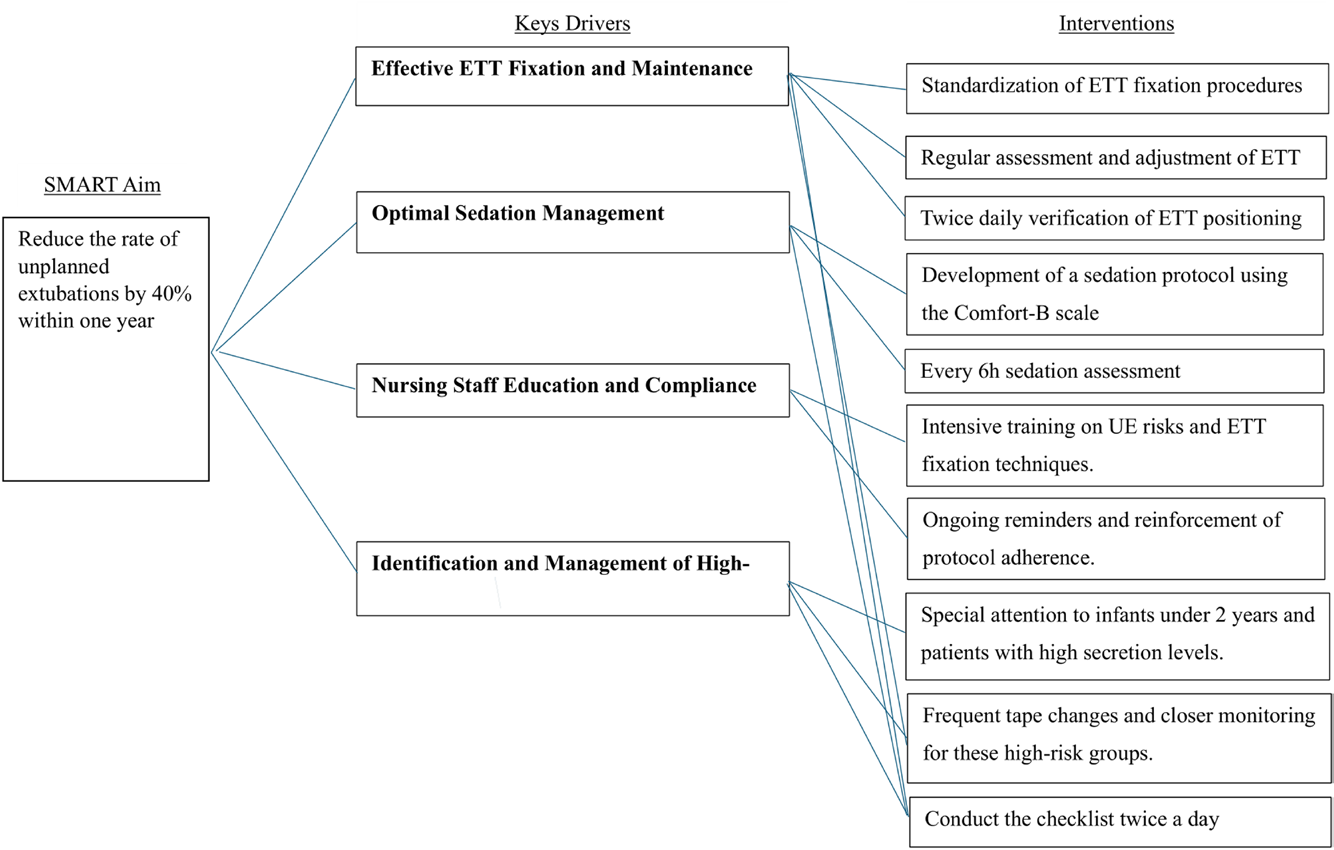
Key driver diagram: Outlines he aim, four key main drivers, and project interventions to reduce UEs.

**Fig. 2.**
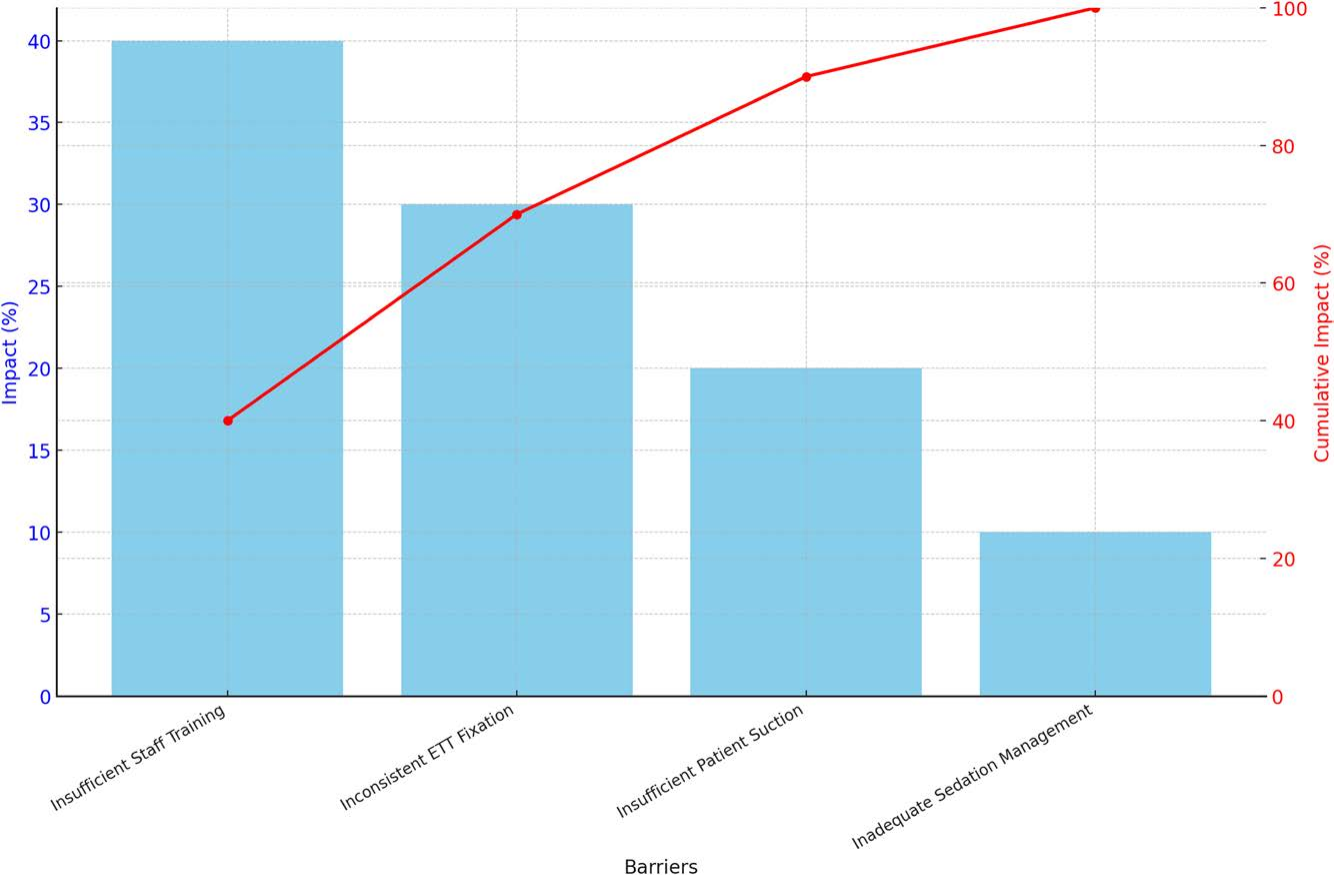
Pareto chart: 4 main barriers to project implementation were found during the training period: insufficient nursing staff training, inconsistent ETT fixation, insufficient patient suction, inadequate patient sedation.

The QI initiative was driven by a SMART aim to reduce the rate of UEs by 40% within 1 year. A key driver diagram (Fig. 1) was developed based on 6 months of baseline data analysis and on published risk factors and interventions known to decrease UE rates in a pediatric population.^7^ The key driver diagram included the use of a Pareto chart to identify critical barriers (Fig. 2). The main drivers identified were:

1. Adequate ETT fixation and maintenance:
  a. Standardization of ETT fixation procedures.
  b. Regular assessment and adjustment of ETT security.
  c. Twice daily verification of ETT positioning.
2. Optimal sedation management:
  a. Development of a sedation protocol using the Comfort-B scale.
  b. Regular (Q 6H) sedation assessment to prevent over- or undersedation.
3. Nursing staff education and compliance:
  a. Intensive training on UE risks and ETT fixation techniques.
  b. Ongoing reminders and reinforcement of protocol adherence.
4. Identification and management of high-risk patients:
  a. Special attention to infants younger than 2 years and patients with high secretion levels.
  b. Every 12-hour tape changes and closer monitoring for these high-risk groups.

Following the development of the key driver diagram, we used plan-do-study-act (PDSA) cycles to iteratively refine and adapt these interventions. The PDSA cycles allowed us to:

- First cycle: Address inconsistencies in ETT fixation among floating nurses by implementing standardized protocols and training.
- Second cycle: Improve sedation management through sedation implementation and regular assessments, adjusting based on feedback.
- Third cycle: Provide continuous staff education and compliance monitoring through training and mentorship, addressing the challenges of staff turnover and floating nurses.

### Nursing Training and Education

To enhance adherence to UE prevention strategies, we conducted structured nursing training sessions focused on ETT fixation techniques, sedation assessment using the Comfort-B scale, and UE prevention best practices. Training included interactive bedside coaching, small-group discussions, and periodic refresher courses integrated into regular team meetings. Float nurses received targeted orientation to ensure practice consistency.

### Compliance Monitoring and Feedback Sessions

Given the absence of formal compliance audits, we implemented structured feedback sessions as an alternative monitoring strategy. These sessions occurred weekly and were led by a senior pediatric intensivist and nursing team leaders. They included bedside discussions and group meetings, where staff reflected on protocol adherence, identified barriers, and proposed real-time solutions. Key topics included ETT fixation practices, sedation management, and challenges in protocol implementation.

Staff participation was encouraged through an open, nonpunitive approach, and feedback was incorporated into ongoing QI adjustments. These discussions served as an adaptive compliance monitoring tool, reinforcing best practices despite resource constraints.

The standardized protocol used for reducing UEs is available as Supplemental Digital Content 1. (**See Supplemental Materials, Supplemental Digital Content 1**, which describes standardized protocol for reducing UEs, http://links.lww.com/PQ9/A649.)

### Study of the Intervention

**Fig. 3.**
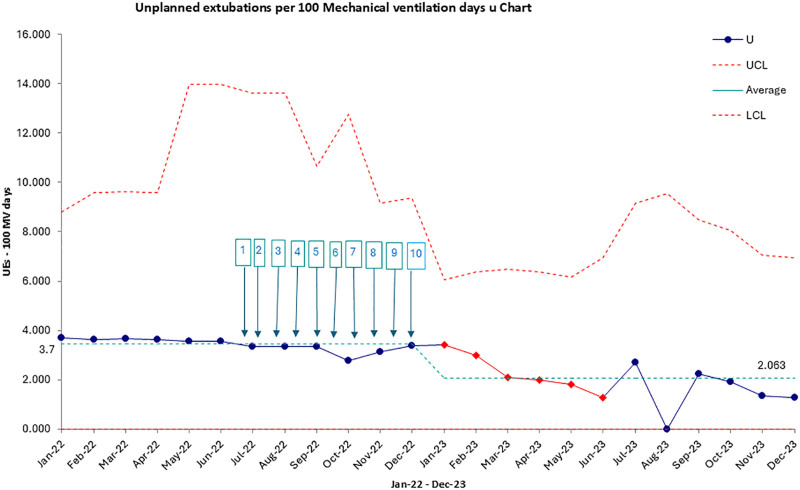
SPC U chart of UEs per 100 MV days. This U chart illustrates the overall reduction in UE rates per 100 MV days after the implementation of the QI interventions from January to June 2022. 1: Intensive training on UE risks and ETT fixation techniques; 2: standardization of ETT fixation procedures; 3: twice daily verification of ETT positioning; 4: regular assessment and adjustment of ETT; 5: training on sedation protocol using the Comfort-B scale; 6: every 6 hours sedation assessment; 7: special attention to infants under 2 years and patients with high secretion levels; 8: frequent tape changes and closer monitoring for these high-risk groups; 9: conduct the checklist twice a day; 10: ongoing reminders and reinforcement of protocol adherence. MV, mechanical ventilation.

An interrupted time series design was used to assess the impact of the interventions. We conducted the study in three phases:

1. Baseline assessment (January 2022–June 2022):
  a. Collection of UE incidence data.
  b. Identification of key drivers using Pareto and fishbone analysis.
2. Implementation of interventions (July 2022–December 2022):
  a. The interventions were implemented in phases between January and June 2022, as different elements of the QI initiative were introduced based on feedback and identified needs. We did not track the individual effects of each intervention as they were introduced. Instead, the study was designed to evaluate the cumulative impact of the entire QI initiative on reducing UEs.
  b. The key driver diagram (Fig. 1) identified the critical areas to address, and interventions such as the standardization of ETT fixation, sedation management protocols, and ongoing staff education were rolled out throughout this period.
3. Postintervention evaluation (January 2023–December 2023):
  a. Assessment of UE rates using a Statistical Process Control (SPC) chart. The SPC chart (Fig. 3) shows the overall reduction in UE rates after the full implementation of these interventions.

### Measures

- Primary outcome measure: Rate of UEs, defined as the number of UEs per 100 days of mechanical ventilation.
- Process measures: We tracked compliance with the protocol components by monitoring adherence to standardized ETT fixation, sedation management, and nursing education sessions. Compliance with ETT fixation was assessed through twice-daily verification of tube security and documentation in patient charts. Sedation management adherence was evaluated by tracking the percentage of patients assessed using the Comfort-B scale every 6 hours, as per protocol. Additionally, all training sessions were documented, and attendance logs were maintained to ensure staff participation. Real-time compliance of these process measures was assured as noted above, though the specific compliance numbers were not tracked over time.

### Analysis

An SPC U chart was utilized (Excel QI macros) to monitor the rate of UEs per 100 ventilation days over time. The baseline period was used to establish the initial control limits. Following implementing the QI interventions (phase 2), the UE rates were tracked to detect special cause variation, indicating a statistically significant shift in the process.

Centerline (CL): The initial centerline was calculated based on the average UE rate during the baseline period.

Control limits (UCL and LCL): The upper and lower control limits were established at ±3 SDs from the CL, representing the expected range of normal variation.

Shift Detection: CL shifts were determined according to standard SPC rules for special cause variation; specifically runs of 8 or more consecutive points on one side of the centerline or any points outside the control limits.

### Ethical Considerations

We obtained ethical approval for the study from the local ethics committee (FB.12/2022), with a waiver for parental consent.

### Reporting Standards

We prepared this article following the Standards for Quality Improvement Reporting Excellence 2.0 guidelines to ensure comprehensive and transparent reporting of the QI initiative.

## RESULTS

During the study period, 110 patients were included. The median age was 3 months (1.5–7.75 mo), ranging from 10 days to 11 years and 7 months. The median duration of mechanical ventilation was 8 days (interquartile range 5–15 d), ranging from 1 to 170 days. The total ventilation days amounted to 1,337 days.

During the baseline period (January to June 2022), 23 patients received mechanical ventilation, with a median duration of 6 days (interquartile range 3–13 d), resulting in a total of 416 ventilation days and 15 UEs occurred (UE rate of 3.62 per 100 ventilation days).

Following the implementation of the QI interventions, 87 patients received mechanical ventilation during the postintervention period (January 2023–December 2023) with a median duration of 7 days, totaling 921 ventilation days, with 19 UEs (UE rate of 2.06 per 100 ventilation days). Patient characteristics during the 2 study periods were comparable (Table 1).

**Table 1.** Patients Characteristics

	Before Phase (First Semester 2022)	After Phase (2023)	*P*
Number (n)	23	87	
Age (mo)	9.6 (1.5–39.4)	4.4 (1.6–21.2)	0.825
Weight (kg)	8.5 (3.9–15.7)	6 (4.2–10.7)	0.567
Boys (%)	16 (69.5)	51 (58.6)	0.594
Premature delivery (n)	3 (13)	15 (17)	0.733
Birth weight (kg)	3.3 (3–3.7)	3.4 (2.8–3.8)	1.000
PIM 3	18.4 ± 20	15.7 ± 22.7	0.740

Comparison of patient characteristics before (1st semester 2022) and after (2023) the QI initiative. Data are shown as median (interquartile range) for continuous variables, number (%) for categorical variables, and mean ± SD for PIM 3 scores. *P* values indicate the significance of differences between the 2 phases. PIM, pediatric index of mortality.

A total of 12 structured nursing training sessions were conducted, covering standardized ETT fixation, sedation monitoring, and UE prevention strategies. Training formats included bedside coaching (1-on-1), small-group workshops, and scheduled refresher meetings. Additionally, compliance with key interventions was monitored throughout the study period. Twice-daily ETT fixation verification was documented in 87% of ventilated patients, and sedation assessments were performed in 78% of eligible cases. In addition to informal bedside observations and chart audits, weekly feedback sessions provided a structured compliance monitoring approach. Over the intervention period, 42 sessions were conducted, attended by pediatric intensivists, nursing leaders, and bedside staff. These sessions reinforced adherence to ETT fixation protocols, sedation monitoring, and overall UE prevention strategies. Staff reported that the sessions helped clarify expectations, address challenges in real-time, and improve team communication, contributing to improvements in protocol adherence.

Implementing the QI interventions resulted in a 42.7% reduction in UE incidence (*P* = 0.015). The SPC U chart (Fig. 3) illustrates the rate of UEss per 100 ventilation days over the study period, divided into the baseline period (January 2022–June 2022) and the postintervention period (January 2023–December 2023). During the baseline period, the UE rate fluctuated around the CL of 3.62 UEs per 100 ventilation days, with control limits set at ±3 SDs. All data points during this period remained within the control limits, indicating a stable but high UE rate.

Following the implementation of QI interventions in July 2022, the chart shows a significant downward shift in the UE rate during the postintervention period, with the new average at 2.06 UEs per 100 ventilator days and several consecutive data points consistently below the centerline. This indicates a statistically significant reduction in UEs, achieving the SMART aim of reducing UEs by more than 40% within 1 year. The absence of points outside the control limits postintervention suggests that the data were improved and the system was stable.

Overall, the SPC U chart suggests that the QI interventions were associated with a sustained improvement in UEs, which is 1 marker of PICU patient safety. This sustained improvement highlights the potential for long-term success in implementing structured QI initiatives in resource-limited settings, such as those with staffing variability, equipment shortages, and inconsistent training availability.

### Unintended Consequences and Balancing Measures

During the implementation of the QI initiative to reduce UE, the increased focus on lowering UEs led to heightened vigilance among nursing staff, contributing to stress and anxiety levels. Nurses reported feeling pressured to adhere to the protocols strictly. The implemented weekly debriefing sessions revealed that although initial stress levels were high, continuous team discussions and feedback mechanisms helped alleviate anxiety over time. By the end of the intervention period, staff reported greater confidence in implementing the standardized protocols.

## DISCUSSION

UEs pose a significant risk in PICUs, with potential consequences including increased morbidity, prolonged ventilation, and longer hospital stays. Our study demonstrated a substantial reduction in UE rates after implementing a targeted QI initiative, leading to enhanced patient safety. Despite significant resource constraints, such as staffing variability, equipment shortages, and inconsistent availability of trained personnel, we achieved a 42.7% reduction in UE rates, demonstrating the adaptability and effectiveness of these interventions even in a resource-constrained environment.

We believe our success was driven by staff commitment, teamwork, and continuous reinforcement of best practices. Regular training, bedside coaching, and weekly debriefing sessions helped sustain adherence and maintain momentum, even with frequent staff turnover. Although formal compliance tracking was not implemented, adherence was informally monitored through chart audits, direct observations, and feedback discussions. The high participation in training and consistent use of twice-daily ETT fixation checks suggested strong compliance. Future initiatives should incorporate systematic compliance tracking to further strengthen QI efforts.

This significant reduction in UEs observed in our study underscores the effectiveness of targeted QI interventions in a resource-limited setting. PDSA cycles were integral to achieving these results, allowing us to iteratively adapt interventions in response to specific challenges faced in our setting. Although our results align with studies conducted in more developed healthcare environments, both in pediatric and neonatal intensive care, such as those by Tripathi et al,^8^ Klugman et al,^9^ and Mahaseth et al,^10^ our study provides insight into the unique challenges and successes of implementing such interventions in a developing country like Tunisia. Our hospital, serving as a regional referral center, faces challenges that are more pronounced in resource-limited settings like Tunisia. These include inconsistent access to specialized equipment, frequent shortages of trained staff, and consistent reliance on float nurses, who may not have consistent experience in pediatric critical care. These factors differ from the more stable staffing and resource availability in high-income countries. These differences can contribute to a lower baseline rate of UEs and smoother implementation of QI interventions in more resource-replete countries.^11^ Despite these significant challenges, our QI initiative achieved important improvements in UE rates.

Our study’s success highlights the importance of adapting QI interventions to the specific context of the healthcare environment. Based on reported risk factors of UEs,^12–15^ the standardization of ETT fixation procedures, improved sedation management, and focused staff training were crucial to achieving the observed reductions in UEs. Additionally, using SPC charts provided a robust quantitative method for tracking outcomes and ensuring that observed changes were not due to random variation but rather associated with our implemented interventions.^16^ Although formal compliance tracking was not implemented, intervention effectiveness was supported by observable practice changes. ETT fixation became a routine bedside priority, sedation assessments were conducted more consistently, and staff engagement in training sessions remained high. These changes aligned with the reduction in UEs, suggesting that process improvements translated into measurable clinical outcomes.

Additionally, continuous adaptations ensured feasibility in our setting. Training was integrated into daily workflow to accommodate staff turnover, floating nurses received targeted bedside coaching, and protocols were simplified to enhance adherence. These strategic modifications helped sustain improvements despite resource limitations.

We believe this report provides valuable insight into how driving QI changes can occur and be adapted in a resource-limited healthcare environment, thus broadening the existing medical literature on this topic.

Despite these promising results, our study has limitations. First, this was a single center, and the improvements accomplished locally may not be generalizable to other institutions. Second, the lack of a randomized control group and potential variations in staff training and patient populations may have influenced the outcomes. Although the Pareto chart displayed key factors contributing to UE, these factors were primarily derived from the literature rather than specifically investigated within our patient population. Rather than conducting a separate analysis for local factors, we focused on commonly reported risk factors in the broader pediatric intensive care setting. Identifying and addressing local risk factors could potentially yield even better results. Third, although compliance tracking was not formally recorded, adherence was reinforced through structured feedback sessions. Although these provided real-time monitoring and practice adaptation, future studies should incorporate objective compliance audits to strengthen QI assessments. Finally, the relatively short follow-up period limits our ability to assess the long-term sustainability of the intervention’s effectiveness. Future research should focus on the long-term impact of QI interventions and consider a multicenter approach to generalize findings across diverse PICU settings.

## Conclusions

Our study contributes to the growing body of evidence supporting the effectiveness of targeted QI initiatives in reducing the incidence of UEs in PICUs. Our observed reduction in UE rates suggests the important role of standardized protocols, including proper ETT fixation, sedation management, and structured nursing training pertaining to standardized ETT fixation and sedation management in a resource-limited environment. Continuous efforts to refine these interventions and address identified risk factors are essential for further improving outcomes in this vulnerable patient population. To sustain these gains, we plan to maintain regular staff training, periodic audits, and providing continuous feedback. Additionally, ongoing debriefing sessions will address staff stress and workload to ensure the protocols remain effective.

## Supplementary Material


