# Supplementary material for: Enhancing Safety in Mechanical Ventilation: A Quality Improvement Initiative Targeting Unplanned Extubations in a Tunisian PICU
Source: Pediatr Qual Saf. 2025 Apr 2;10(2):e805. doi: 10.1097/pq9.0000000000000805 (PMC11964382; doi:10.1097/pq9.0000000000000805)
Supplement: Supplementary file 1 [file pqs-10-e805-s001.pdf]

Standardized Protocol for Reducing Unplanned Extubations (UEs)

1. Overview of Standardized Protocols

A. Effective Endotracheal Tube (ETT) Fixation and Maintenance

- **Objective:** Ensure secure and stable positioning of the ETT to minimize accidental extubations.
- **Procedures:**
  - **Standardization of ETT Fixation:**
    - Use of commercially available ETT fixation devices to secure the tube.
    - Apply appropriate tension to the fixation device to prevent movement without causing trauma.
  - **Regular Assessment:**
    - Conduct assessments of ETT security during each nursing shift (every 12 hours) and after any patient repositioning.
    - Use a checklist to document ETT position and security status.
  - **Verification of Positioning:**
    - Verify the ETT depth and position twice daily and after any manipulation of the patient.

B. Optimal Sedation Management

- **Objective:** Prevent agitation and ensure adequate sedation levels for intubated patients.
- **Procedures:**
  - **Sedation Protocol Development:**
    - Implement the Comfort-B scale for sedation assessment.
    - Sedation goals: Maintain a sedation score of …. on the Comfort-B scale.

- **Regular Sedation Assessment:**
    - Perform sedation assessments every 6 hours.
    - Adjust sedation levels based on assessment results to avoid over- or under-sedation.

C. Nursing Staff Education and Compliance

- **Objective:** Enhance nursing staff knowledge and adherence to protocols to reduce UEs.
- **Procedures:**
  - **Training Sessions:**
    - Conduct initial training for all nursing staff on UE risks and ETT fixation techniques.
    - Provide training materials, including visual aids and written protocols.
  - **Ongoing Education:**
    - Schedule monthly refresher training sessions.
    - Use case studies to discuss challenges and solutions related to UEs.
  - **Compliance Monitoring:**
    - Use checklists to monitor adherence to protocols and provide feedback to staff.

D. Identification and Management of High-Risk Patients

- **Objective:** Provide additional monitoring and care for high-risk patients to prevent UEs.
- **Procedures:**
  - **Risk Assessment:**
    - Identify infants under 2 years and patients with high secretion levels as high-risk.
    - Maintain a list of high-risk patients to ensure close monitoring.

- **Enhanced Monitoring:**
  - Implement more frequent monitoring for high-risk patients (at least every 2 hours).
  - Document interventions and observations in the patient's electronic medical record.

2. <u>Nurse Orientation Process</u>

- **Objective:** Ensure that all nursing staff, especially new hires, or floating nurses are familiar with the standardized protocols.
- **Procedures:**
  - **Initial Orientation:**
    - New nurses will undergo a comprehensive orientation program that includes training on the standardized protocols.
    - Provide a manual that includes all protocols and procedures.
  - **Mentorship Program:**
    - Assign experienced nurses as mentors to new staff for at least the first month.
    - Mentors will provide hands-on training and support in implementing protocols.
  - **Evaluation:**
    - Conduct evaluations after the first month to assess new nurses' understanding and compliance with the protocols.
    - Provide feedback and additional training as needed.
